# Design rules for a tunable merged-tip microneedle

**DOI:** 10.1038/s41378-018-0028-z

**Published:** 2018-10-22

**Authors:** Jungeun Lim, Dongha Tahk, James Yu, Dal-Hee Min, Noo Li Jeon

**Affiliations:** 1 School of Mechanical and Aerospace Engineering, Seoul National University, Seoul, 08826 South Korea; 2 Division of WCU Multiscale Mechanical Design, Seoul National University, Seoul, 08826 South Korea; 3 Institute of Advanced Machinery and Design, Seoul National University, Seoul, 08826 South Korea; 4 Interdisciplinary Program for Bioengineering, Seoul National University, Seoul, 08826 South Korea; 5 Department of Chemistry, Seoul National University, Seoul, South Korea

## Abstract

This publication proposes the use of an elasto-capillarity-driven self-assembly for fabricating a microscale merged-tip structure out of a variety of biocompatible UV-curable polymers for use as a microneedle platform. In addition, the novel merged-tip microstructure constitutes a new class of microneedles, which incorporates the convergence of biocompatible polymer micropillars, leading to the formation of a sharp tip and an open cavity capable of both liquid trapping and volume control. When combined with biocompatible photopolymer micropillar arrays fabricated with photolithography, elasto-capillarity-driven self-assembly provides a means for producing a complex microneedle-like structure without the use of micromolding or micromachining. This publication also explores and defines the design rules by which several fabrication aspects, such as micropillar dimensions, shapes, pattern array configurations, and materials, can be manipulated to produce a customizable microneedle array with controllable cavity volumes, fracture points, and merge profiles. In addition, the incorporation of a modular through-hole micropore membrane base was also investigated as a method for constitutive payload delivery and fluid-sampling functionalities. The flexibility and fabrication simplicity of the merged-tip microneedle platform holds promise in transdermal drug delivery applications.

## Drug delivery: self-assembly of microneedles

The fabrication of merged-tip microneedles by elasto-capillarity-driven self-assembly is investigated in photocurable polymers, revealing design parameters for their potential use in drug delivery. Microneedles can be used for delivery of drug payloads by perforating the skin. However, existing needle fabrication processes typically rely on expensive approaches such as etching, embossing, and molding. Now, a team from Seoul National University led by Noo Li Jeon, explore design parameters for fabrication by elastic-capillary-driven self-assembly: photolithography is used to fabricate closely spaced polymer micropillars, the tips of which crosslink to form a microneedle. A number of microneedle geometries are studied, for different UV-curable polymers, suggesting the possibility of their use in drug delivery.

## Introduction

The use of an elasto-capillary interaction for the self-assembly of micropillar array structures has many potential applications^1–3^. Previous efforts to document the elasto-capillary self-assembly process have succeeded in assembling microscale helical and joined multi-pillar photopolymer arrays^4^, as well as a variety of nanoscale carbon nanotube formations^5^.

In a recent development in the field of transdermal drug delivery, microneedles have been developed to provide a cost-effective, eco-friendly, less painful, and easier-to-use alternative to conventional hypodermic needles^6^. Recent efforts to directly integrate lyophilized pharmaceutical payloads with microneedle platforms have already demonstrated their ability to avoid the logistical burdens of the cold chain^7^. Microneedle types and fabrication methods vary widely, but most device types suffer from a heavy reliance on expensive and slow microscale etching^8^, embossing^9^, or molding^10^ techniques, or have other challenging limitations. In general, microneedle classifications include the solid, dissolving, and hollow types^11^.

The simplest microneedle type, solid microneedles, are sharp microstructures capable of penetrating the stratum corneum. Solid microneedles can be used to deliver drug payloads by a topical application to the perforated skin after the application of the microneedle array, or by direct administration through a coating of the drug on the needle array itself^12,13^. In the former, bare microneedle arrays are applied to the skin, which penetrate the stratum corneum of the outer dermis in order to increase the surface permeability to the substances applied on top of the perforation site. In the latter, microneedle arrays are first coated with a drug solution, either through dipping^14^ or spraying^15^. The drug solution coating is then fixed to the microneedle array for injection. Upon penetration, the drug coating on the microneedle array dissolves and disperses into the surrounding tissue. Hollow-type microneedles are fabricated with an empty channel, and can be used to continuously release controlled doses of drugs from a reservoir^16^ or to draw bodily fluids for sampling^17^. High failure rates in the demolding steps and additional treatment processes such as etching^18^ and laser machining^19^ pose significant challenges in solid, coated, and hollow microneedle fabrication. In some cases, the drug payload can be used as the substrate for the microneedle itself. Microneedles fabricated from a solidified water-soluble drug substrate can be engineered to penetrate the skin and dissolve, leaving no biohazardous sharp waste^20^. Dissolvable microneedles are generally either molded^21–23^ or are pulled and cut into shape, which is referred to as drawing lithography^24,25^. This method requires harsh fabrication conditions, which may degrade or destroy pharmaceutical substances. Efforts to fabricate solid-type microneedle arrays from simple photolithography^26^ in an attempt to circumvent microscale molding and etching have resulted in weak monopillars that are unable to endure the mechanical stresses of transdermal penetration, rendering them unsuitable as microneedle platforms. Due to the commercial scale drug delivery aspirations of microneedle technology, reliance on highly expensive and specialized fabrication techniques is both costly and counterproductive.

It is the aim of this publication to propose an optimized fabrication method that uses the photolithographic process with defined design rules for a self-assembled merged-tip microneedle (MTM) array platform for potential drug delivery applications. As a potential transdermal microneedle system, much consideration was needed in addressing the material and chemical requirements. The mechanical properties, such as the Young’s modulus, strength, hardness, and toughness (to facilitate skin penetration), as well as the chemical properties, such as biocompatibility, needed to be satisfied to produce a viable medical needle. Therefore, this publication identifies several biocompatible polymer candidates, including poly (ethylene glycol) diacrylate (PEG-DA) and high-modulus biocompatible photopolymer (HBP), with rigorous testing employed in order to establish design parameters with respect to the array dimensions, component pillar quantities, and pillar array configurations. The resultant data on the fracture test profiles, cavity payload capacities, assembly success rates, penetration profiles, and more have been defined and annotated for reference.

As a microneedle platform, the merged tip has the potential to be a functional addition to existing microneedle types. MTMs can be fabricated in various dimensions, shapes, and compositions to desired specifications in accordance with any number of desirable parameters. Characteristics, such as the aspect ratio and cavity volume, can be precisely set through the modification of the fabrication conditions. Pattern variables, such as the pillar shape (circular, concave, triangle, and convex), pillar array arrangements, and size, coupled with later-stage fabrication variables, such as the ultraviolet (UV) exposure time, are integral to the customization process. Substrates can be selected to confer additional functionality to the proposed MTM platform, as MTMs can be fabricated on top of the pores of a porous PEG-DA through-hole membrane substrate to allow for a continuous release with only the addition of a few fabrication steps. Therefore, certain MTM platforms can be readily constructed by manipulating the design of film mask, material, and substrate.

## Results and discussion

### MTM array

**Fig. 1:**
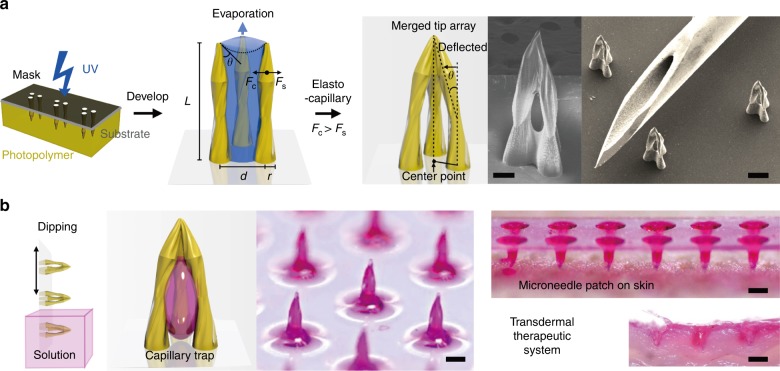
Overview of MTM Fabrication and Deployment. **a** Photolithographic process for merged-tip microneedle fabrication with PEG-DA resin, with a schematic diagram of PEG-DA micropillar interactions with forces during the evaporation of the developer. An SEM image of the merged-tip microneedle manufactured with three circular micropillars with a micropillar radius of 75 μm and an interpillar distance of 300 μm (left) (scale bar: 100 μm). The other SEM image is the array of the microneedles next to a tip of a 25-gauge needle for scale (right). The scale bar indicates 300 μm. **b** Demonstration of the transdermal therapeutic system from trapping the liquid in the microneedle to injecting the microneedle into the skin

An overview of the elasto-capillarity-mediated MTM fabrication process is shown in Figure S1. A PET film base is applied on top of a solution of pre-crosslinked PEG-DA, and a patterned film mask is applied over the PET film base. The film mask–PET film–PEG-DA solution stack is then exposed to UV light through the mask for 45 s (dose: 450 mJ/cm^2^). The PEG-DA solution initiates crosslinking where it is exposed to the UV light through the transparent patterns of the field film mask, forming solid, parallel PEG-DA micropillar arrays anchored perpendicular to the PET film base. Following UV exposure, the nascent parallel PEG-DA micropillar arrays are immersed in a developer (in this case, 70 wt% ethanol with 30 wt% of deionized (DI) water) and then allowed to dry (for 5 min in a 70 °C oven). The purpose of the solvent immersion is twofold. First, the solvent washes out excess un-crosslinked PEG-DA from the micropillar array; second, the solvent also serves as a means by which the parallel micropillars coalesce into a joined point via an elasto-capillary interaction. For solvent-mediated coalescence to occur, micropillars must be over a critical threshold height directly related to the pillar pattern shape, radius, and distribution density. The forces involved in the merging tip process can be analyzed through the relationship between the evaporation-induced elasto-capillary forces, and the resistant standing forces in the merged micropillar arrays (Fig. 1a). The micropillar standing force, *F*_s_ quantified as:

$$F_{\mathrm{s}}\sim \frac{{Er^4d}}{{h^3}}$$

where *r* is the micropillar’s radius, *h* is the height, *d* is the interpillar distance, and *E* is the Young’s modulus. The elasto-capillary contractile force *F*_c_ exerted on the micropillars by the solvent meniscus is quantified by:

$$F_{\mathrm{c}}\sim \frac{{\gamma r^2{\mathrm{cos}}^2\theta }}{d}$$

where *γ* denotes the interfacial tension of the volatile developer, *θ* is the contact angle, and all other variables are carried over from the standing force equation^27^. Micropillar array designs, dimensions, and arrangements are mainly controlled by adjusting the film mask pattern. Variations in the interpillar distances (*d*) also typically result in structural variation. In the given figure, two micropillar arrays of identical dimensions and shapes were fabricated with different interpillar distances. The array with the larger interpillar distance resulted in a lower aspect ratio and a higher cavity volume than the array with the smaller interpillar distance, demonstrating that interpillar gap distances can also be utilized as a means of customizing MTM designs to suit a specific function. The MTM array can be applied to the transdermal therapeutic system, as shown in Fig. 1b.

### Design of the MTM array

**Fig. 2:**
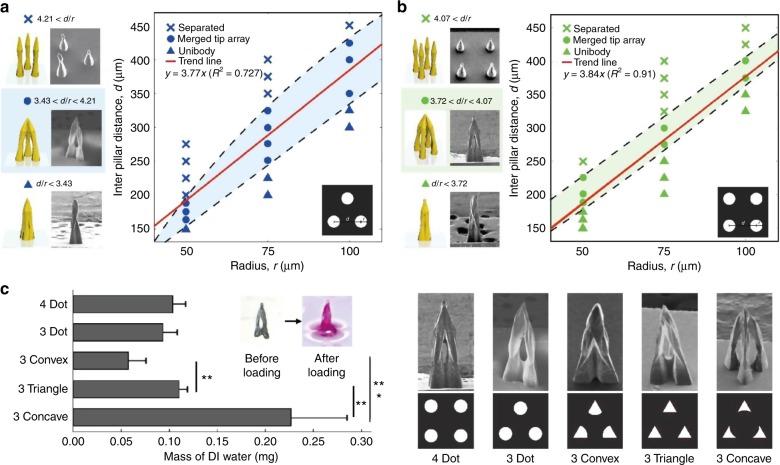
Design Rules for Tip Merging. Structural phase diagram for merged-tip microneedle fabrication without integrating the micropillars into a single body and separating the micropillars (▲: uni-body, ●: merged tip, ×: separated); SEM images and illustration of elasto-capillarity-driven patterning for each phase with the respective number of micropillars demonstrated above the graph. In addition, the range of the distance-to-radius ratio corresponding to each phase is indicated under the images, while the equation of the design rule for constructing MTMs is on the graph. **a** Three circular patterns (scale bar: 200 μm) and **b** four circular patterns (scale bar: 150 μm). **c** Mass of DI water to be trapped in the cavity of a single MTM, which is constructed using three concave-, three triangle-, three convex-shaped patterns, three circular, and four circular, respectively. Error bars represent standard deviations (*n* = 5). Significance levels were set at **P < 0.01, ***P < 0.001. The design of the template mask corresponding to the respective shape of pattern (top) and SEM images of MTMs manufactured using the film mask for each type of pattern (bottom) are demonstrated. The scale bar indicates 200 μm

To quantify the relationships between MTM design variables, MTMs were fabricated in two broad experimental schemes; the first scheme fabricated three-pillar arrays with varying radii and interpillar gap distances (Fig. 2a), while the second scheme fabricated four-pillar arrays with the same experimental format (Fig. 2b). The relationships between micropillar radii and interpillar distances on the merged-tip fabrication process were qualified and sorted under uni-body, merged tip, and separated conditions (Fig. 2a, b). Uni-body arrays were defined as tips in which the micropillars merged too closely to allow for any significant cavity volume, which generally resulted from the tips being too close together relative to their radius. Separated arrays were defined as tips that failed to merge at all, usually due to a prohibitively wide interpillar distance proportional to the micropillar radius. A linear regression was conducted on the data concerning the relationship between the pillar radius and the interpillar distance with respect to a successfully merged tip. The resultant trend line showed that for three-pillar arrays the average merged-tip distance-to-radius ratio (as denoted by the trend line’s slope) is 3.77. To be specific, in the case of the three micropillar array, if the distance-to-radius ratio is between 3.43 and 4.21, a merged tip is formed, while a coalesced single body is constructed under 3.43 and the separated micropillars are patterned over 4.21. For four-pillar arrays, the average successful distance-to-radius ratio was found to be 3.84. Specifically, in the case of four micropillar array, if the distance-to-radius ratio is between 3.72 and 4.07, the micropillars merge into a sharp tip with an open cavity, whereas a uni-body is formed under 3.72 and the micropillars are separate over 4.07. In both cases, the average distance-to-radius ratios in the successfully merged-tip arrays were close to 4. In a structure with three successfully merged-tip pillars, the content of the trapped solution increases with an increasing interpillar distance and radius. On the other hand, it was confirmed that the content of the trapped solution in the structure with four successfully merged-tip structures did not change considerably, even when the interpillar distance and radius increased. As demonstrated, manipulation of the micropillar radius and interpillar distances play a critical role in successful fabrication, and the difference in the constituent pillar amounts within a given array can influence the tolerance thresholds for a successfully merged tip. Moreover, the MTM arrays showed a range of variation in trapped solution resulting from differences in the micropillar radius and interpillar distances (Figure S2). The MTM height was also shown to increase proportionally to the micropillar radius. For each of the sampled three-pillar arrays (radii of 50, 75, and 100 μm), the overall MTM heights were found to be 436, 945, and 1242 μm, respectively. In four-pillar arrays with radii of 50, 75, and 100 μm, the average overall MTM heights were observed to be 678, 1046, and 1560 μm, respectively.

To verify that the design rule matches the elasto-capillarity equation relationship, the experimental results were compared to the theoretical value from the equivalence of the standing force and capillary force equations^1,27^. The data from the three micropillar PEG-DA MTMs, which each had a 50 μm radius and a height of 436 μm, were applied to the standing force and capillary force equations. The MTMs were constructed for a PEG-DA solution to be exposed to UV light for 45 s, and the PEG-DA micropillars were developed using 70 wt% ethanol with 30 wt% of DI water; the average Young’s modulus *E* was 151.7 MPa, and the interfacial tension *γ* was 25.01 mN/m.

$$F_{\mathrm{s}} = \frac{{3\pi Er^4v}}{{4h^3}}$$

In the standing force equation, *v* is the horizontal distance of the micropillar tip from its bottom, which is *d*/2 under the merged-tip condition.

$$F_{\mathrm{c}} = \frac{{2\pi \gamma r^2{\mathrm{cos}}^2\theta }}{u}$$

In the capillary force equation, *u* is the displacement between the two micropillar tips, which rapidly decreases as the micropillars are tilted, with *u* equal to *d* in the initial state. Since the micropillars do not stay upright, the maximum capillary force immediately before the two micropillars contact each other is estimated to be three orders of magnitude higher than the initial capillary force. Therefore, according to the presumption above, we can acquire the equation of the interpillar distance when the standing force equals the capillary force.

$$d = 1120\sqrt {\frac{{35\gamma r{\mathrm{cos}}^2\theta }}{{3E}}}$$

By applying *θ* = 60 °C as the typical contact angle to the interpillar distance equation, we can acquire the distance between the two micropillars *d* = 173.7 μm when the standing force and the capillary force are in equilibrium as *F*_c_ = *F*_s_ = 1.13 μN. According to the design rule established from the experimental results, the range of the interpillar displacement to form merged tip was 167.5–187.5 μm, which includes the theoretical value of the distance in the force equilibrium state. As a result, the experimental design rule is comparable to the theoretical force relationship.

### Cavity control of the MTM array

In addition to the previously quantified micropillar radius, interpillar distance, and pillar number, shapes can also influence the outcome of a merged tip. To quantify the relationship between the micropillar shapes on the resultant MTMs, arrays in varying shapes were fabricated with varying dimensions. The tested array shapes were three dot, four dot, three concave, three triangle, and three convex. The array dimensions tested consisted of micropillar radii of 50, 75, and 100 μm, where both the diameter and interpillar space were kept equal to one another. It was observed that a micropillar radius of 75 μm and an interpillar distance of 300 μm resulted in the most consistent successful tip merging across all tested shapes (Figure S3). Using a micropillar array with a radius of 75 μm and an interpillar distance of 300 μm as a standard, all tested array shapes were tested for their interior capacity. The MTM arrays were fabricated in 3 × 2 batches per shape, weighed, immersed in DI water, and weighed again in order to determine the trapped mass of DI water. The liquid can be readily trapped in the cavity of the MTM after being dipped in the liquid (Fig. 4a). The total difference in weight per 3 × 2 batch was divided by 6 in order to determine the average capacity of a single given array. The resultant values by array shape were logged and analyzed with an analysis of variance (Fig. 2c). Based on the cavity analysis, it was determined that the micropillar shape played an important role in determining the MTM cavity volume. From the highest to lowest trapped volume, the tested shapes are as follows: concave, triangle, four dot, three dot, and convex. In particular, the cavity volumes of the MTMs with the concave-, triangle-, and convex-shaped micropillars showed critical differences (*P* < 0.01 or <0.001 for each pair). Furthermore, by dividing the trapped mass of the DI water for each MTM by the density of water, the volume of the cavity can be easily calculated in a single MTM basis (Figure S4). Assuming that the trapped fluid of the MTM cavity directly relates to the dosage volume of a given MTM, the design shapes of the constituent micropillars in a given array can be modified to customize the dosage volume for drug delivery.

### Axial fracture force of the MTM array

**Fig. 3:**
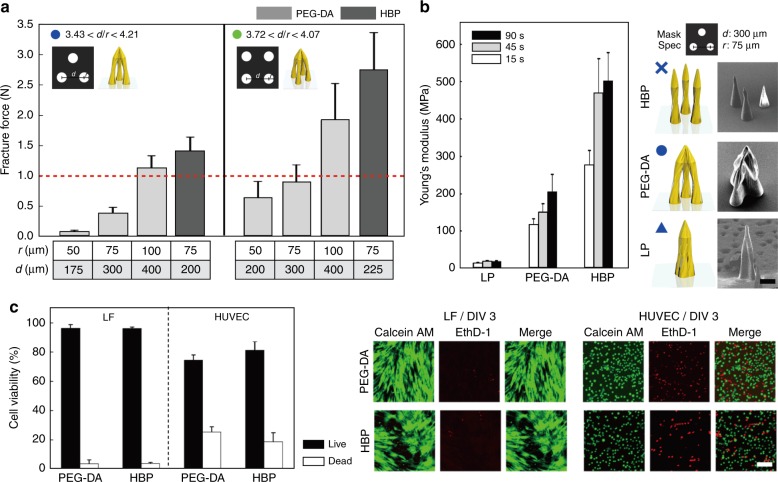
Fracture Force and Biocompatibility Validation. **a** Axial load fracture force test of a single MTM constructed with two biocompatible polymers (HBP and PEG-DA) for varying array patterns (three- and four-pillar arrays) and pattern radii (50, 75, and 100 μm). **b** Young’s modulus value of biocompatible polymers (LP, PEG-DA, and HBP) by exposure time (15, 45, and 90 s), under the same elasto-capillarity-driven patterning conditions and film mask designs with three different biocompatible polymers (LP, PEG-DA, and HBP). The scale bar indicates 200 μm. The illustration and SEM images of the patterning in each polymer are located on the right side of the graph. **c** Graph of cell viability derived from the viability test of human umbilical vein endothelial cell (HUVEC) and lung fibroblast (LF) cultured on PEG-DA photopolymer and high-modulus biocompatible photopolymer (HBP). The scale bar is 100 μm

To be viable, the MTM array must be able to penetrate the 10–20 μm stratum corneum without breaking. The MTM array penetration capabilities were assessed by a multifunction adhesion scratch test system. In the fracture force test, an axial compression load was applied to the MTM array to determine the relationship between the fracture force and the structure of MTM arrays, which was fabricated with a circular-patterned film mask by adjusting variables such as the number of the patterns, radius of the patterns, and number of MTMs per array. A total of three different types of arrays were tested in this fracture test: three (3 × 1), six (3 × 2), and nine (3 × 3) MTMs in each array. As shown in Fig. 3a, as the pattern radius and micropillar thickness increased, the fracture force tended to increase. In addition, as the number of circular patterns and MTMs in the array increased, which implies more micropillars to support the MTM, the fracture point also increased. In general, the minimum average fracture point required for penetration of the stratum corneum without fracture was 0.1 N/ea^28,29^. The minimum axial load fracture force for PEG-DA MTMs was 0.32 ± 0.04 N for a 3 × 1 array fabricated with three circular patterns of 50 μm radius and the maximum axial load fracture force was 16.37 ± 2.59 N for a 3 × 3 array fabricated with four circular patterns with a 100 μm radius (Figure S6). This indicates that all of the tested MTM array types were suitable for skin penetration. In assessing the axial fracture force profile of individual PEG-DA microneedles, the minimum fracture force found was 0.08 ± 0.025 N fabricated with three circular patterns with a 50 μm radius and the maximum axial load fracture force was 1.91 ± 0.09 N (in 4 × 100 μm radius circular patterns) (Fig. 3a). All but one MTM array types tested were found to be suitable for skin injection, namely, the 3 × 50 μm radius single microneedle fracture test. The data suggest that the MTM arrays tested in this fracture force analysis have adequate strength to withstand injection into the skin.

As seen in the graph, MTMs fabricated using HBP had a higher fracture point. Specifically, the axial fracture force of MTMs manufactured with HBP was higher than the force of MTMs composed of PEG-DA with the same micropillar number and radius (Fig. 3a).

**Fig. 4:**
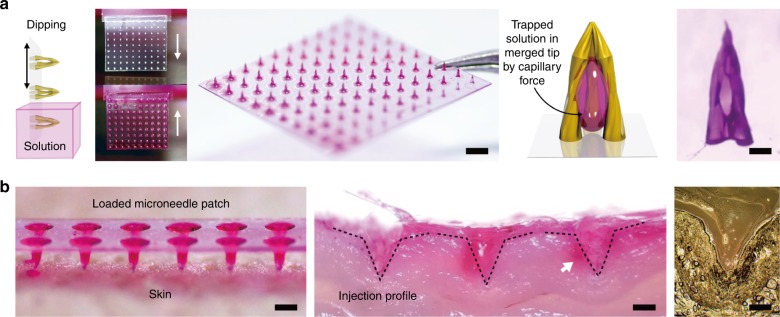
MTM Loading and Injection. **a** Schematic illustration of the microneedle loading process. Photograph of a 10 × 10 MTM array dipped in rhodamine B solution (scale bar: 2000 μm) with before and after photographs. A schematic diagram of the rhodamine B solution trapped in the MTM interior cavity with an optical microscopy image of a single loaded MTM array (scale bar: 200 μm). **b** Photograph of the MTM array loaded with the rhodamine B solution on the skin (scale bar: 1000 μm) and cross section illustrating the injection profile of the MTM (scale bar: 500 μm). The optical microscopy image of the cross section for a single MTM injection profile (scale bar: 300 μm)

One of MTMs fabricated with PEG-DA, which had an appropriate fracture force for injection, was selected for injection testing. The test array was loaded with a solution of rhodamine B (0.1 wt% in DI water) and applied to a section of shaved rat dorsal skin. The MTM array successfully penetrated the stratum corneum without any mechanical failure, demonstrating suitability for injection with a payload (Fig. 4b).

### Mechanical properties and biocompatibility of the MTM array

According to the given equation, as the Young’s modulus *E* increases, the standing force *F*_s_ also increases. The relationship between *E* and *F*_s_ was confirmed experimentally by substituting PEG-DA with low-modulus photopolymer (LP) and HBP. The particular chemical composition of UV-curable resin of LP, PEG-DA, and HBP is indicated in Figure S5 and the biocompatibility of PEG-DA and HBP is demonstrated in Fig. 3c. Micropillar arrays of LP, PEG-DA, and HBP were separately fabricated by being subjected to elasto-capillarity-driven patterning under 45 s UV exposure using three circular pores with 75 μm radii and an interpillar distance of 300 μm on the film mask. The young’s modulus of LP, PEG-DA, and HBP after UV exposure for 45 s are 18.7, 151.7, and 469.6 MPa, respectively, which is prone to increase by increasing the UV exposure time as shown in Fig. 3b. The more elastic PEG-DA micropillar arrays, however, successfully self-assembled into merged configurations, while the more rigid HBP arrays did not. On the other hand, in case of LP, the micropillar arrays are fully merged without an open space in the center since the standing force is too low to endure the capillary force driving the micropillars to the center, which lead to a collapse (Fig. 3b). To overcome the limitation of microneedle dimensions, we fabricated MTMs using HBP by increasing the exposure time (100 s) with a film mask design of PEG-DA MTMs, which leads to an elongated height of the micropillar. To apply a more stiff and biocompatible polymer to the elasto-capillarity-driven patterning, a HBP is utilized in the MTM fabrication.

### Continuous drug delivery MTM array system

**Fig. 5:**
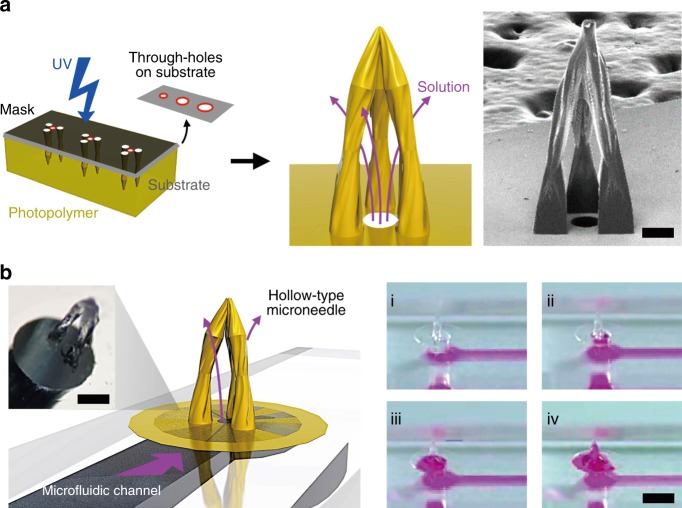
Hollow Type MTM Fabrication and Loading. **a** Schematic diagram of the photolithographic fabrication process and liquid delivery system of the hollow MTM with an SEM image of a three triangular pillar array design. The scale bar indicates 200 μm. **b** Illustration of the solution delivery through the microfluidic channel-based pores on the through-hole membrane into the cavity of the MTM and the optical microscopy image of the system. Sequential photographs of the rhodamine B solution flow through the pore of the through-hole PEG-DA membrane by time (steps i–iv). The scale bar indicates 2000 μm

Hollow-type microneedles are a conventional type of microneedle used for continuous flow in various applications, such as the continuous drug release of fluid extraction. An MTM can gain hollow-type continuous flow functionality by incorporating a porous basement substrate or a through-hole membrane, called a hollow MTM (Fig. 5a). The overall fabrication process of a hollow MTM array, shown in Figure S7, is nearly identical to the process for non-perfusable MTM arrays, with the exception of the addition of pattern alignment with the porous basement membrane substrate, an adhesive poly(dimethylsiloxane) (PDMS) layer, and additional required exposure time. The substrate pores at the center of each MTM cavity connect directly to a basement substrate-embedded channel. As part of the manufacturing process, a layer of PDMS was applied to the bottom of the film mask to ensure a good conformal contact between the template mask and the PEG-DA substrate, as well as to mitigate the PEG-DA solution leakage through the membrane pores during the exposure process. Due to the permeable gas characteristics of PDMS, oxygen within the PDMS membrane inhibits free radical-induced PEG-DA polymerization. To compensate for the crosslinking inhibitory effects of the trapped oxygen, the exposure time was increased by 100 s to a total of 140 s (dose: 1400 mJ/cm^2^) for proper micropillar formation^30^. Through this comparatively simple fabrication process, a hollow MTM can be readily fabricated in less time and at a lower cost than with the conventional manufacturing processes. The hollow MTM array can function as both a hollow microneedle as well as an MTM, allowing for both single-dosage as well as constant release dosage mechanisms. Hollow MTM arrays can be formed on PDMS microfluidic chip channel openings for irrigation-like functionality. As seen in Fig. 5b and Movie S1, a rhodamine B solution is injected through the PDMS microfluidic channel at a rate of 0.5 mL/min and is shown perfusing into the MTM cavity on the other side. As demonstrated, hollow MTM arrays can be used to constitutively flow fluid, indicating a potential application for continuous drug delivery.

## Materials and methods

### Fabrication of MTM array

As the MTM array substrate, a flexible and clear polyester film is pretreated on both sides for enhanced adhesion to the UV curing resin (thickness: 100 μm, skyrol V7200, SKC, Korea). A UV-curable PEG-DA solution consisting of a mixture of 97 wt% (by mass) PEG-DA (Mn 250, Sigma-Aldrich, viscosity 15–35 cP) pre-polymer, and 3 wt% 1-hydroxy-cyclohexyl-phenyl-ketone (Irgarcure 184, BASF) as a photoinitiator is prepared as the material for the needles. UV exposure is conducted with a 10 mW/cm^2^ UV aligner (Shinu MST, South Korea) with an *i*-line filter. The resultant micropillars are developed with a solution of 70 wt% ethanol and 30 wt% DI water. The MTM array was cured under UV light for 12 h (UV wavelength: 250–400 nm, dose: 100 mJ/cm^2^). The photolithographic process on the PET film is carried out using the fabrication method described by Kang et al.^31^. The overall fabrication process is shown in Figure S1.

### Live dead assay

The live/dead viability test is conducted using a kit with calcein AM (0.5:1000) and ethidium homodimer-1 (0.5:1000) for mammalian cells. The media for human umbilical vein endothelial cell (Lonza) is Endothelial Growth Medium (EGM-2, Lonza), and the media for normal human lung fibroblast (Lonza) is Fibroblast Growth Medium (FGM-2, Lonza).

### Mechanical properties

The mechanical modulus is analyzed using the universal test machine (Instron 3343) at a strain rate of 10 mm/min. The test specimen films are fabricated and cut into 5 mm × 50 mm strips, each with a grip size of 10 mm. Thin films of LP, PEG-DA, and HBP are exposed to UV light for 15, 45, and 90 s. The blue hill 3 generated the test reports.

### Mechanical fracture force

The axial compression force required for the mechanical fracture of the MTM arrays is measured using a multifunction adhesion scratch test system (model AST210, Neoplus). Each of the three MTM arrays (3 × 1, 3 × 2, and 3 × 3) are fixed on the metallic plate and compressed against a flat block of the testing machine, applying a steady force at a rate of 0.2 mm/s. Force and displacement data are recorded, and the fracture force at the point where the MTM broke is measured using these data.

### Skin insertion

A Sprague-Dawley rat is prepared for the skin insertion test. A 3 × 2 MTM (four circular pillars with 100 μm radius) array is loaded with a solution of rhodamine B (Sigma) and applied to the shaved dorsal skin of the rat. The MTM array is removed from the skin after 10 min of penetration. The cross-sectional photograph of the skin at the region of the injection shows the penetration profile of the MTM array without any evidence of mechanical failure. All experiments are conducted according to the guidelines of the Institutional Animal Care and Use Committee of Seoul National University.

### Fabrication of continuous drug delivery MTM array

A porous PEG-DA membrane with a 100 μm-diameter pore size and a 2 mm inter-pore space is produced as a substrate. The porous PEG-DA membrane can be simply prepared using a rapid and high-throughput through-hole membrane fabrication process^32^. A 30 μm-thick layer of PDMS (Sylgard 184, Dow Corning, USA) is applied to the bottom of the film mask to prevent PEG-DA leakage and to ensure conformal contact between the PEG-DA substrate and the mask. A UV-curable PEG-DA solution consisting of a mixture of 97 wt% of PEG-DA (Mn 250, Sigma-Aldrich, viscosity 15–35 cP) pre-polymer and 3 wt% of 1-hydroxy-cyclohexyl-phenyl-ketone (Irgacure 184, BASF) as the photoinitiator is prepared. UV exposure is conducted with a 10 mW/cm^2^ UV aligner (Shinu MST, South Korea) with an *i*-line filter for 140 s (dose: 1400 mJ/cm^2^). The resultant micropillars are developed with a solution of 70 wt% and 30 wt% DI water. The MTM array is cured under UV light for 12 h (UV wavelength: 250–400 nm, dose: 100 mJ/cm^2^). The overall fabrication process of the hollow MTM array, shown in Figure S7, is nearly identical to the process for non-perfusable MTM arrays with the exceptions of the addition of pattern alignment with the porous basement membrane substrate, an adhesive PDMS layer, and additional required exposure time. A microfluidic device composed of 100 μm-wide microchannel-patterned PDMS block is integrated with a covered slip glass as a substrate. Before combining the coverslip glass with the PDMS block by treating with air plasma for covalent bonding, the start point of the microchannel is punched with a 1 mm biopsy punch. The single hollow MTM punched out using the 2 mm biopsy punch is covalently bonded on the 1 mm hole via air plasma treatment after aligning the 100 μm-diameter pore of the through-hole membrane with the 1 mm punched hole on the PDMS block.

## Conclusions

This publication proposes the fabrication of a MTM platform using an elasto-capillarity-driven self-assembly of biocompatible photolithographic polymer pillars. The MTM array platform is a highly customizable and modular system capable of accommodating a variety of design specifications to suit specific functional needs. As discussed, the optimal design rules determined for forming merged-tip structures rely on the relative ratio between the interpillar distance and the micropillar radius. Patterned arrays with high micropillar radii coalesce into uni-body structures, while arrays with high interpillar distances tend to remain as separate micropillars. Uni-body arrays, which form a solid composite of component micropillars, possesses a higher degree of mechanical strength than monopillar solid-type microneedles and are available as an alternative. Separated micropillar arrays have already seen many applications in cell and liquid trapping and have many potential uses in tissue culture^33–36^. Within the parameters set by the design rules, MTM arrays can be fabricated with a variety of aspect ratios, dimensions, fluid cavity volumes, shapes, and rigidities by manipulating the size, shape, and number of the constituent array micropillars and by changing the material of the microneedles themselves. MTM arrays can also incorporate modular substrate components, such as a through-hole substrate, to add additional perfusable channel functionalities for potential continuous delivery or constant sampling applications. By varying the shape, number, and dimension of patterns on the template masks, an MTM array with a desired cavity volume can be constructed for a specific purpose of use, enabling the fine manipulation of molecule dosage delivery. In conclusion, MTMs have the potential to be transdermal drug delivery platforms with both single and constant dose capabilities.

## Electronic supplementary material

Movie S1

Supplementary Information
