# Supplementary material for: Design rules for a tunable merged-tip microneedle
Source: Microsyst Nanoeng. 2018 Oct 22;4:29. doi: 10.1038/s41378-018-0028-z (PMC6220166; doi:10.1038/s41378-018-0028-z)
Supplement: Supplementary file 2 — Supplementary Information [file 41378_2018_28_MOESM2_ESM.pdf]

Supporting Information

Design Rules for a Tunable Merged Tip Microneedle

Jungeun Lim[1,2,†], Dongha Tahk[1,2,3,†], James Yu[5], Dal-Hee Min[4] and Noo Li Jeon[1,2,3,5]*

1. School of Mechanical and Aerospace Engineering, Seoul National University, Seoul 08826, South Korea.
2. Division of WCU Multiscale Mechanical Design, Seoul National University, Seoul 08826, South Korea.
3. Institute of Advanced Machinery and Design, Seoul National University, Seoul 08826, South Korea
4. Department of Chemistry, Seoul National University, Seoul, South Korea
5. Interdisciplinary Program for Bioengineering, Seoul National University, Seoul 08826, South Korea.

[†]Equally contributed author, [*]Corresponding author, njeon@snu.ac.kr

* Corresponding author.

E-mail: njeon@snu.ac.kr.

Phone: +82-2-880-7111.

Fax: +82-2-880-7119.

Key words: elasto-capillarity, self-assembly, photolithography, polymeric microneedle

S1. Design Rule Analysis

In order to identify that the design rule matches the elasto-capillarity equation relationship, the experimental result is compared to the theoretical value from the equivalence of the standing force and capillary force equations [1,27]. The data of three micropillars PEG-DA MTM which has 50 μm radius and 436 μm height is applied to the standing force and capillary force equations. As the MTM is constructed for PEG-DA solution to be exposed to UV light for 45 seconds and the PEG-DA micropillars are developed by 70 wt% ethanol with 30 wt% of deionized (DI) water, the average young's modulus E is 151.7 MPa and the interfacial tension γ is 25.01 mN/m.

$$F_s = \frac{3\pi E r^4 v}{4h^3}$$

In the standing force equation, v is the horizontal distance of the micropillar tip from its bottom, which is $d/2$ for merged tip condition.

$$F_c = \frac{2\pi\gamma r^2 cos^2\theta}{u}$$

In the capillary force equation, u is displacement between the two micropillar tips which rapidly decreases as the micropillars are keep tilted and u is equal to d in the initial state. Since the micropillars do not stay upright, the maximum capillary force right before two micropillars contact each other is estimated to be three orders of magnitude of the capillary force at initial state. Therefore, according to the presumption above, we can acquire equation of the interpillar distance when the standing force equals to the capillary force.

$$d = 1120\sqrt{\frac{35\gamma r cos^2\theta}{3E}}$$

By applying $\theta = 60°C$ as the typical contact angle to the interpillar distance equation, we can acquire the distance between two micropillars d = 173.7 μm when the standing force and the capillary force are in equilibrium as being $F_c = F_s = 1.13$ μN. According to the design rule established from experimental results, the range of interpillar displacement to form merged tip is 167.5 μm ~ 187.5 μm, which include the theoretical value of the distance in force equilibrium state. As a result, the experimental design rule is comparable to the theoretical force relationship.

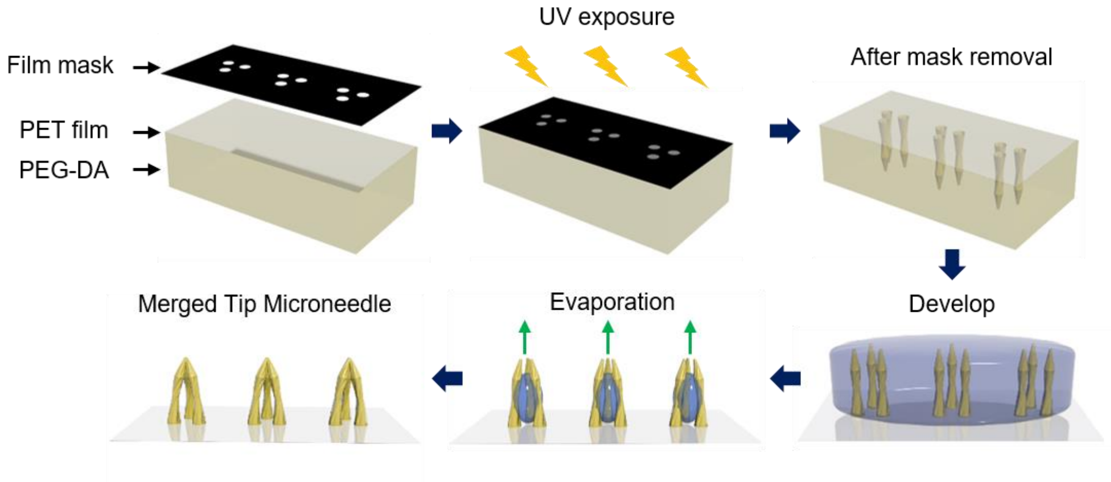

Figure S1. Step-by-step schematic of MTM fabrication using elasto-capillarity and photolithographic process.

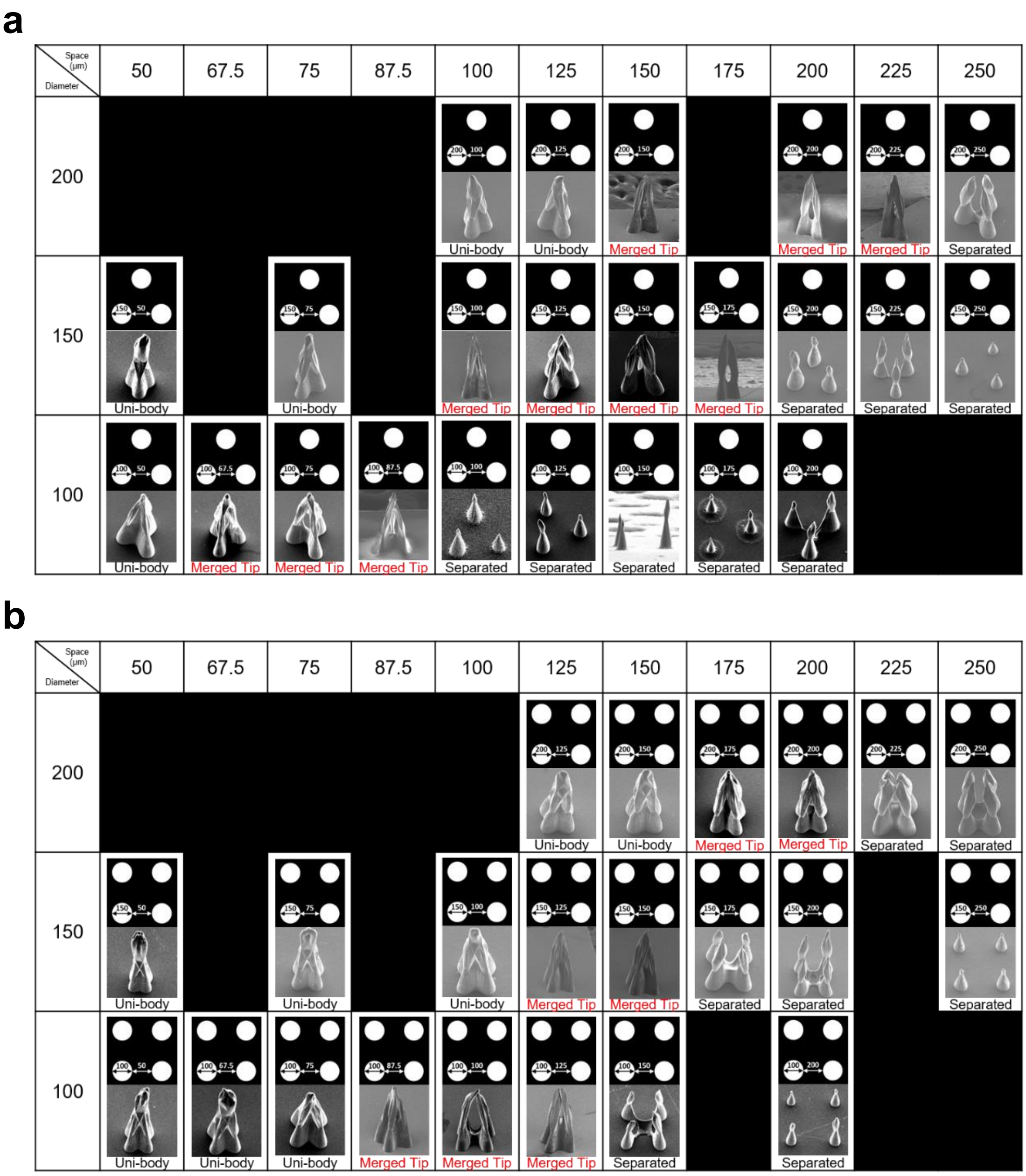

Figure S2. Mask design (upper) and SEM images (bottom) corresponding to respective dimensional condition, as MTM is manufactured with (**a**) three micropillars and (**b**) four micropillars.

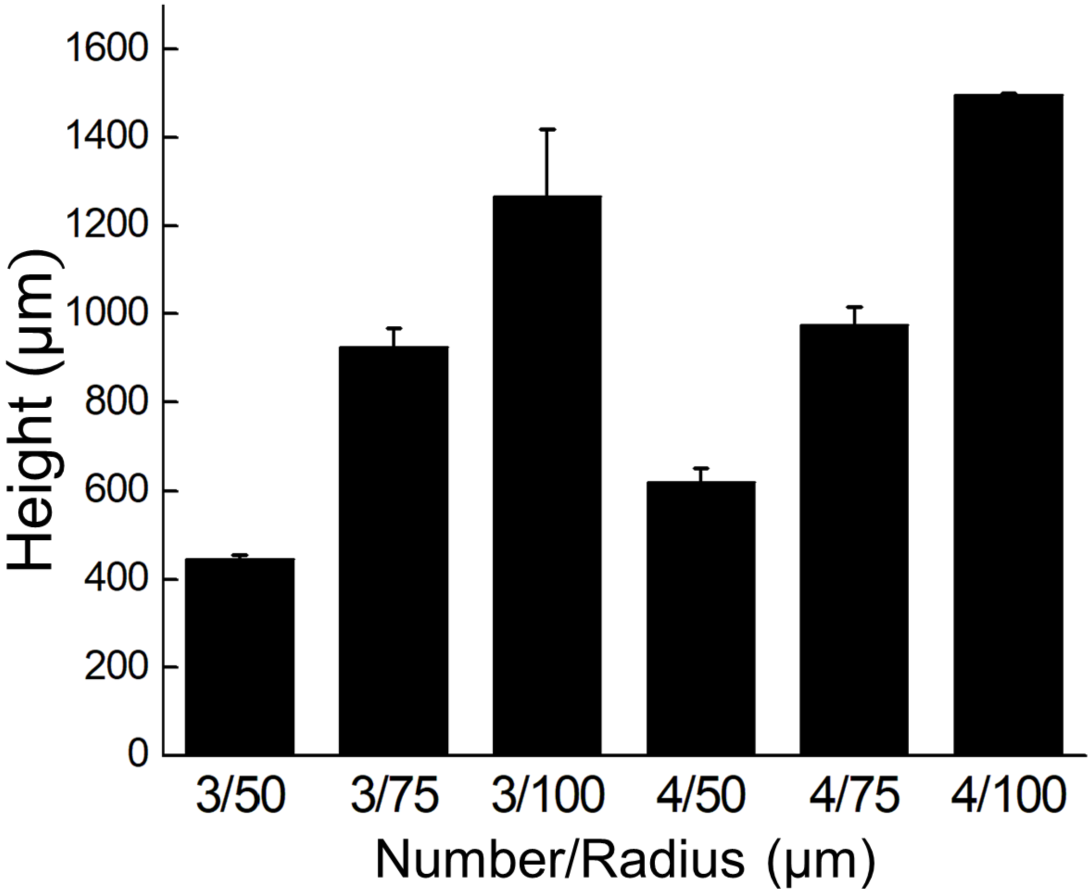

Figure S3. Height of merged tip microneedle arrays fabricated with varying number of the patterns (three and four), radius of the patterns (50 µm, 75 µm and 100 µm). Error bars represent standard deviations (n = 5).

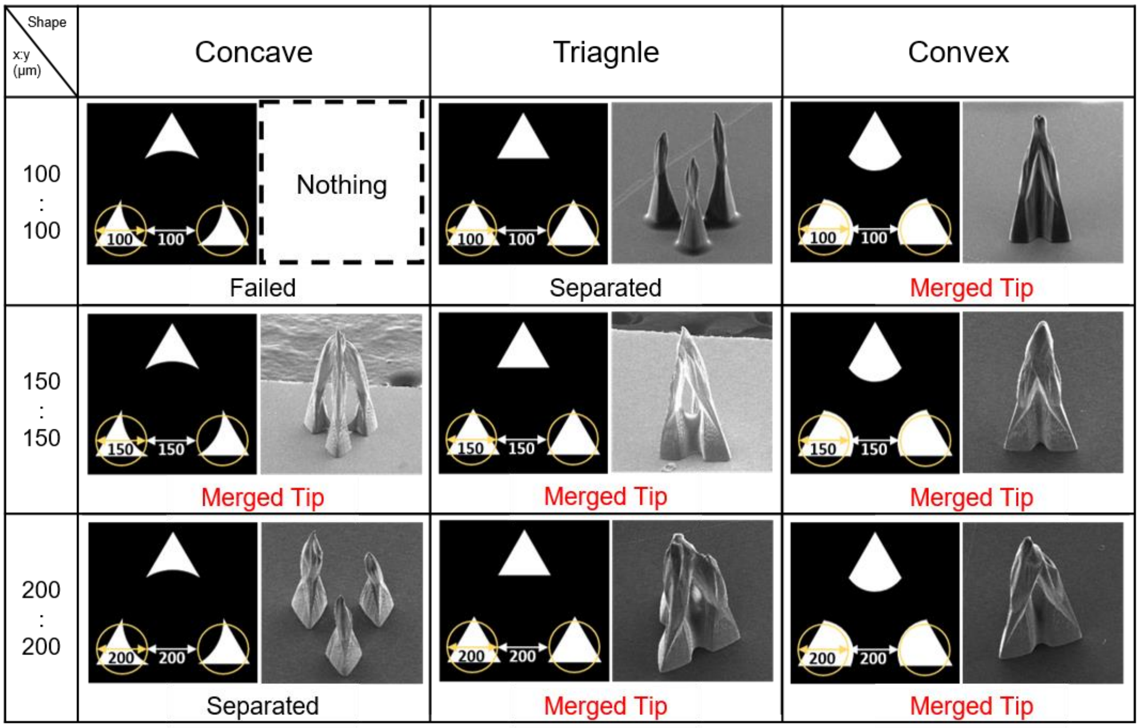

Figure S4. Mask design (left) and SEM images (right) corresponding to respective shape of patterns and dimensional condition, as the MTM are fabricated with three micropillars.

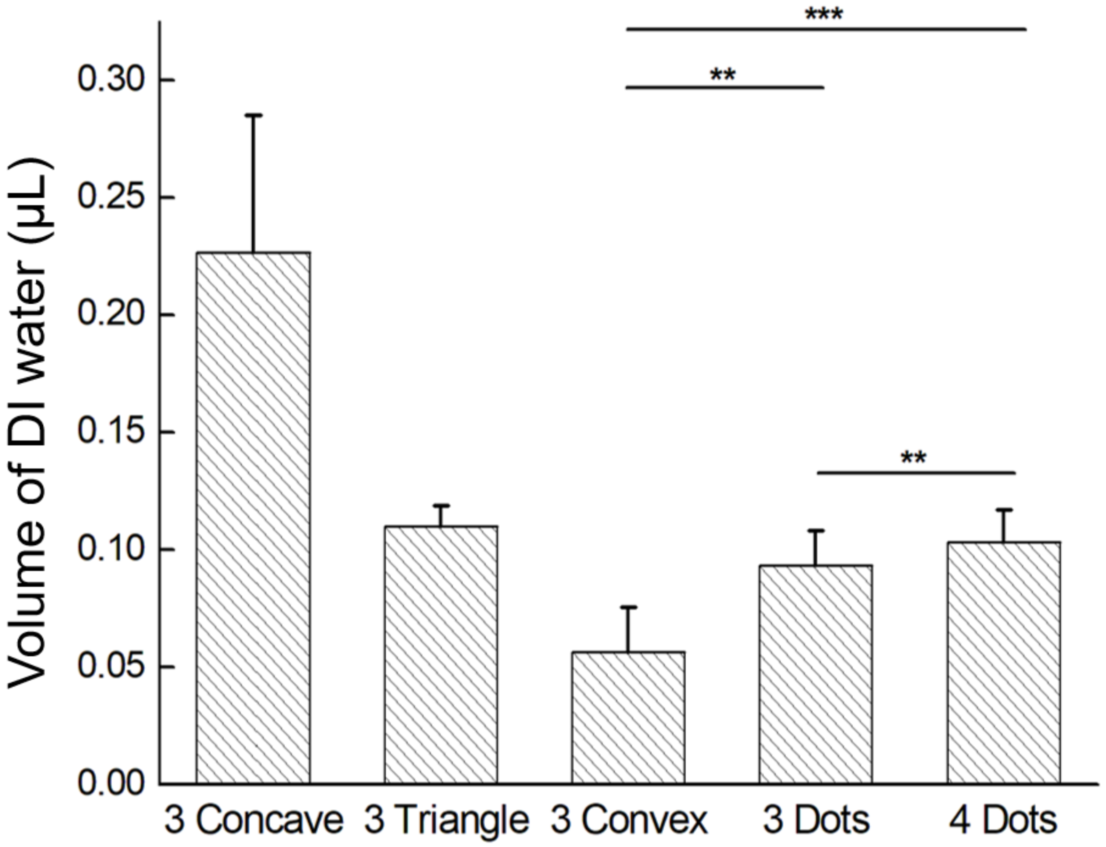

Figure S5. Volume of DI water to be trapped in the cavity of a single MTM, which is fabricated using three concave, three triangle, three convex shaped, three circular and four circular patterns, respectively. Error bars represent standard deviations (n = 5). Significance levels were set at **P<0.01, ***P<0.001. Under the graph, SEM images of merged tip microneedle manufactured using film mask for each shape of patterns and the design of the template mask corresponding to respective shape of pattern are demonstrated.

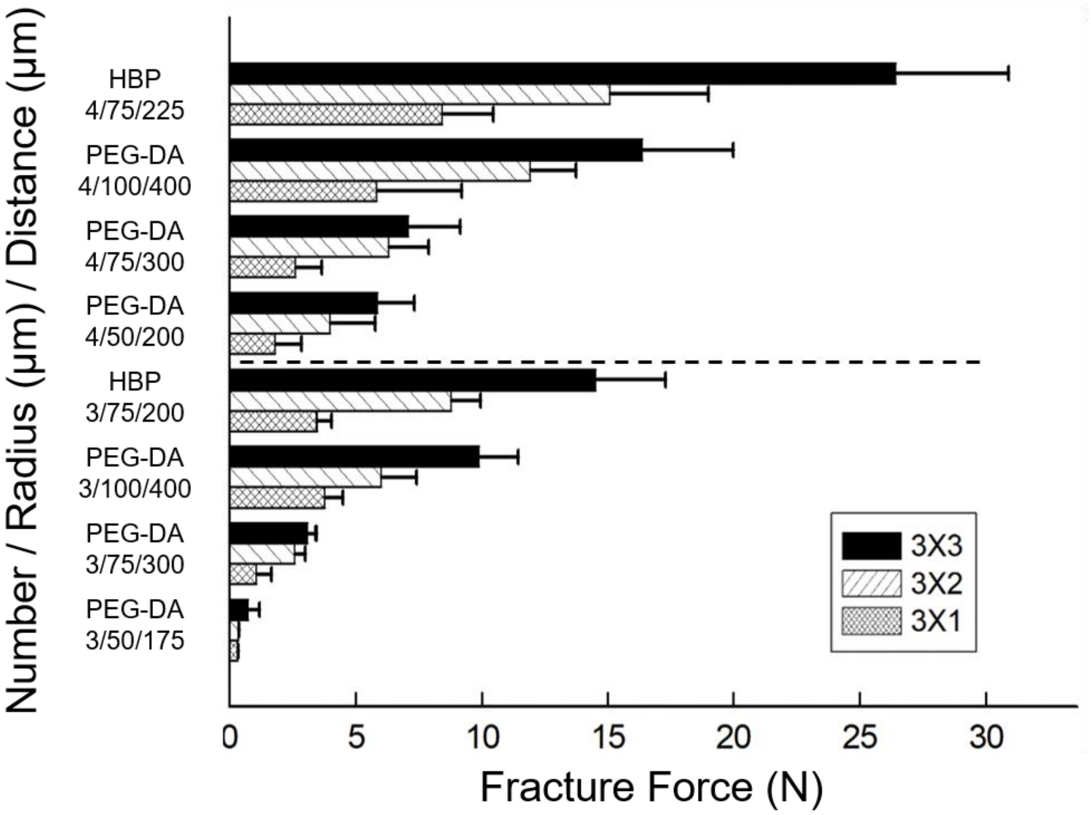

Figure S6. Axial load fracture force test of merged tip structure arrays constructed with two biocompatible polymers (HBP and PEG-DA) for varying number of the patterns (three and four), radius of the patterns (50 μm, 75 μm and 100 μm) and arrays (3 × 1, 3 × 2 and 3 × 3).

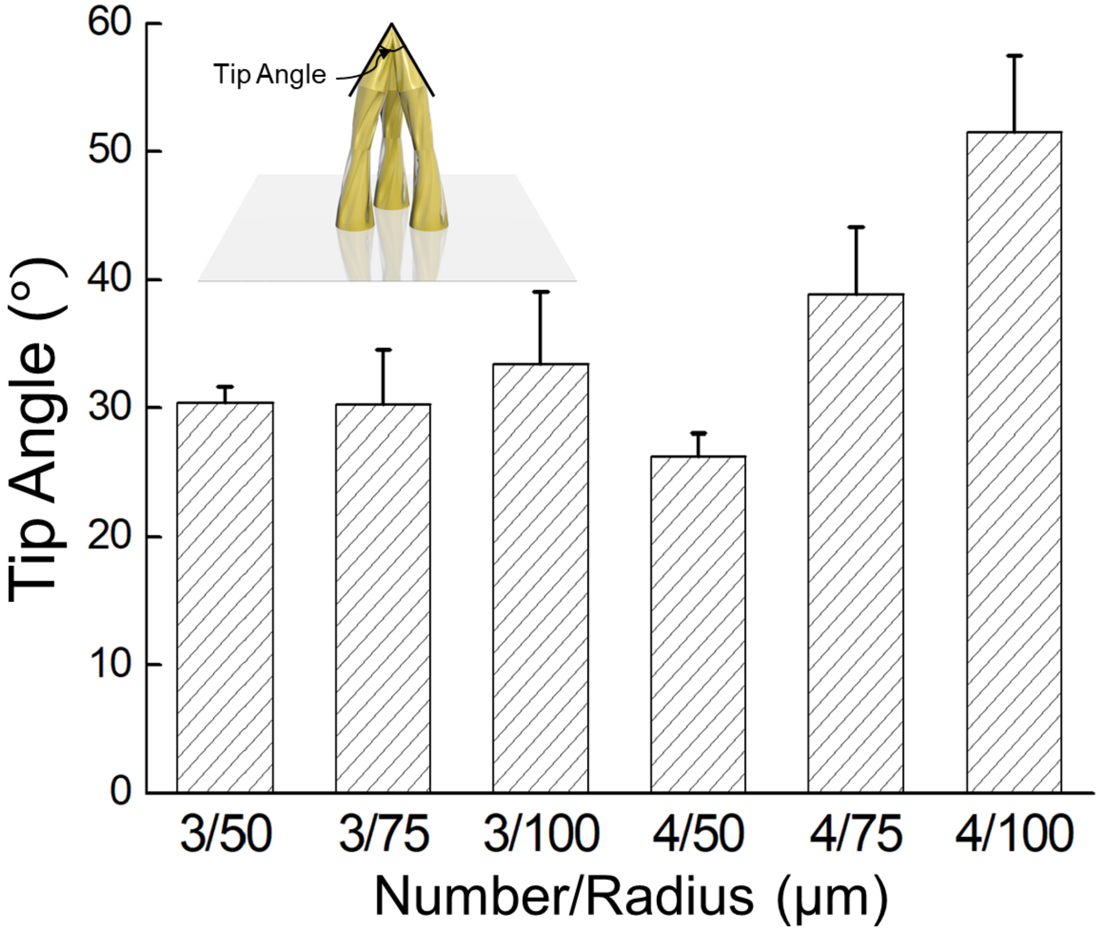

Figure S7. Tip angle of merged tip microneedle arrays fabricated with varying number of the patterns (three and four), radius of the patterns (50 μm, 75 μm and 100 μm). Error bars represent standard deviations (n = 5).

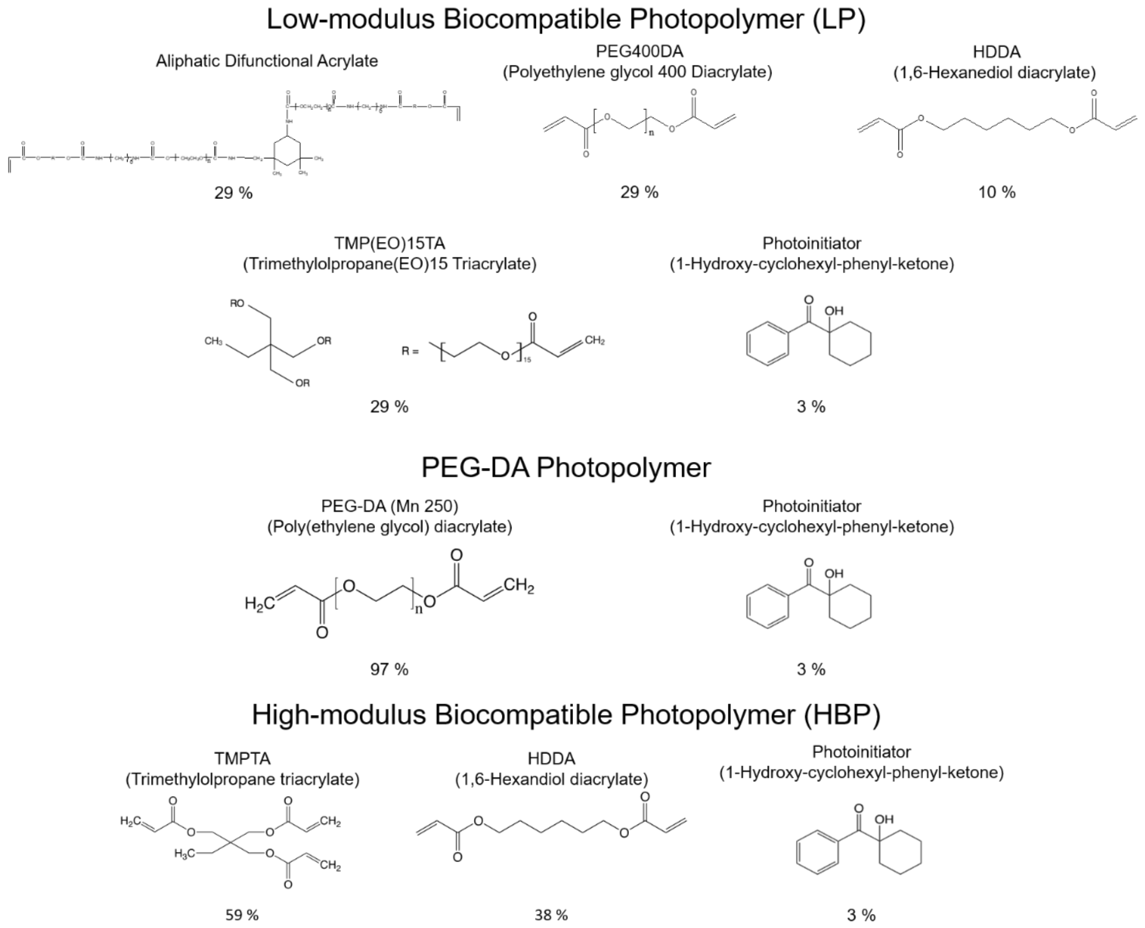

Figure S8. Chemical composition of LP, PEG-DA photopolymer and HBP.

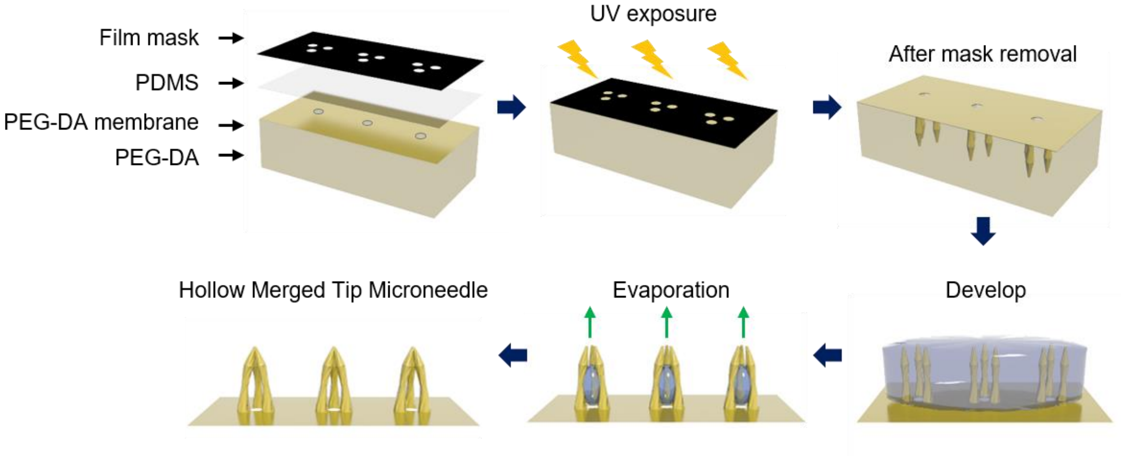

Figure S9. Step-by-step schematic of hollow MTM fabrication using elasto-capillarity and photolithographic process on the porous PEG-DA membrane.
